# The Hospitals of New York

**Published:** 1893-06-24

**Authors:** 


### The Institutional Workshop.

### WITHIN THE HOSPITALS.

### THE HOSPITALS OF NEW YORK.

(By Our Special Commissioner.)

The hospitals of New York may be described in two principal divisions—(*a*) those which are under the city corporation, and (*b*) those supported by endowment or voluntary contributions. In our article to-day we shall deal only with the voluntary hospitals. These voluntary hospitals consist of two classes—*i.e.*, general and special hospitals. The principal general hospitals are the New York Hospital, the Presbyterian Hospital, the St. Luke's Hospital, the Roosevelt Hospital, and Mount Sinai Hospital. It is pleasant to observe that during the last fifteen years, and especially during the last ten years, very marked improvement has taken place in the administration of all these institutions. Notably the character of the nursing and service, and also the sanitary arrangements, show marked efficiency, and they will now compare favourably with some of the best administered hospitals in the world. Each of the great voluntary hospitals admits paying patients, but it is regretable to find the tendency increases to admit more and more free cases everywhere. Having regard to the prosperity of the American people, and their remarkable independence, we are of opinion that the authorities would incur a very grave responsibility if they permitted the continuous extension of the amount of free medical relief unless they are assured, after careful inquiry, that the needs of the really poor impel them to increase the number of free beds theretofore made available for this class of patients. The older nations of the earth are suffering from a system of pauperism so gigantic as to be fatal in many ways to the well-being of the peoples, whilst it works most injuriously to the hard-worked members of the medical profession in many countries. We have always regarded it as one of the glories of the United States that the manly independence which all classes robustly exhibit, has found its proper place in the system under which medical relief is there provided for those who need it. This independence finds expression in the circumstance that at the present time the number of hospital beds to each thousand of the population is materially less in the United States than in any other country. This is so because each man and woman feels it to be an advantage to be attended whenever possible at their own homes by their own medical practitioner. Such a system as this deserves to be built up, encouraged, and strengthened by every intelligent citizen of every class. It would be a calamity if a false desire to give largely in charity were to find its expression in the provision of an ever-increasing number of free medical beds in the general and other hospitals of the United States, unless incontrovertible proof is forthcoming beforehand that such a new departure is imperatively called for by the pressing needs of the poorer population. Having personally visited most of the large centres of population and many agricultural and other districts of the United States, where we have come into close contact with many classes of citizens, we are of opinion that the need for the increase in the number of free beds within the hospitals, and the lessening of the number of pay beds in the general wards, which is now taking place, is not called for on the grounds of humanity, whilst evidence of its wisdom in the best interests of the people is, in our view, altogether wanting. We venture to make these introductory remarks in the hope that they may cause discussion, which, by calling public attention to the important bearings of the proportionate supply of free and pay beds, upon the problem of medical relief, may tend to prevent the rapid changes for the worse which we seem to observe in the systems of admission to the hospitals of the United States. Those who desire to compare the working of American hospitals with those of other countries, so far as the issues here dealt with are concerned, will find abundant material for their purpose in the third volume of "Hospitals and Asylums of the World" (Scribners, New York; The Scientific Press, London).

### The New York Hospital.

This institution has been established upwards of one hundred and twenty years. It is situtated off Fifth Avenue, and occupies an excellent site. It has an income of about 700,000 dols. a year, and, so far as we can judge from the treasurer's report, some £120,000 a year is available for the work of the society. It consists of the hospital in the Bloomingdale Asylum, occupying the grounds extending from 114th to 120th Street, and from Fifth Avenue to the Boulevard; and the House of Relief, 160, Chamber Street, for the temporary care and treatment of emergency cases. The Society also has a farm at White Plains, Westchester County, where they are now erecting a new asylum building, to which the patients will be removed from Bloomingdale in 1894, when the new asylum is to be ready for occupancy. The New York Hospital is the mother of medical education in the United States. At the present day we were informed, that nearly all the members of the resident medical staffs at the principal New York hospitals, are supplied through the medical school attached to this great institution. It has a large and representative medical library, which would be much more useful if arrangements could be made to publish a catalogue of the books it contains, so as to make them more readily accessible than they are at present. The resident staff are magnificently housed in the Administration Building, formerly a mansion, and the Nursing Home, which contains a separate sitting-room for every two nurses, whose bed rooms communicate with it, must greatly add to the comfort and happiness of the nursing staff. At the top of the Nursing Home is a sick bay for the isolated cases of infectious disease, and the view from the roof of the Home presents many attractive features. A portion of the roof is covered by an awning where hammocks are slung, so that the nurses may procure rest in hot weather under the most favourable conditions. We shall have something to say about the system of nursing in another article, but we may here state that the type of nurses employed is very high, their surroundings and opportunities for study are excellent, and the bright and happy faces everywhere met with prove how happy and contented these women workers in reality are.

*The Wards and Offices.*

The most striking feature of the interior of this hospital is undoubtedly the ward floors. These are all tiled, and they have no covering of any kind. The encaustic tiles used are of a cheerful pattern, and the design is on the whole striking and effective. Inquiry elicited the fact that thick bath towels are used at the side of the beds for the patients to stand on when dressing, and that the heating by steam, which is the system employed throughout this institution, causes all feeling of coldness to be obviated. We could not discover any special advantage which the tiles have over wood floors, and we are strongly of the opinion that the latter have many advantages which should prevent the general adoption of tiles for hospital purposes. The wards are rectangular in shape, with cross ventilation, but having been erected at least twenty years they have not the structural advantage by which each bed is cut off from every other bed by a window on each side, and so the evils of end beds, which are here strikingly apparent, would be avoided. This drawback was on the day of our visit made more real by the introduction into some wards of extra beds, a practice difficult to prevent, but always to be condemned. In every other respect the wards were not only satisfactory, but commendable. In appearance they are bright and pleasant, beds, mattresses, and bedding are all of the best, and the patients seemed most comfortable and well cared for. It is but just to say that it would be difficult to find wards in any hospital better kept, more tastefully arranged, or anywhere an administration more perfect as a whole. This is strong praise, but it is well deserved, especially as we found the system permitted the employment in some wards of none but probationer nurses. The charge nurse in one case having been eighteen months in training, and her assistants being merely "capped" nurses, that is, pupils who, having passed through a two months' probation, had been admitted for training. There was an absence of flowers in the wards, which was noticeable to an English eye, but this may have been due to the unusually severe winter and to the time of the year at which our visit was paid. In one respect this hospital presents a structural detail which we think worthy of general imitation. The lavatories and baths are situated in a spacious building at the end and side of each ward. Here there is an abundance of space which affords many advantages, administrative and hygienic, which we had not appreciated until we saw the plan in actual work. All the fittings are of brass or marble, the baths being porcelain. These lavatories were beautifully clean, airy, and sweet, and we think this portion of the buildings of the New York Hospital may fairly claim to be nearly as perfect as it is possible to make it. The slop sinks were similar to those at the London Hospital, but they had both a hot and cold water service, although our experience leads us to hold that no slop sink should be fitted with anything but cold water. The cisterns to the w.c.'s were too small, and the flush sadly defective. We mention this fact because we understand a contrary opinion is held by the persons who supplied them. Any practical person must, however, see that the present flushing arrangements will lead eventually to evils which ought not to be possible at a hospital.

*General Remarks.*

We were shown over the hospital by Miss Sutcliffe, the housekeeper, to whom great credit is due for her energy and success in keeping the hospital so beautifully clean. She has, however, some disadvantages to contend with, the kitchen being too small, and the space devoted to it far too limited for so large an establishment. The laundry, which is at the top of the building, contains obsolete machinery; and as the hospital is in the possession of abundance of funds, we hope no time will be lost in making arrangements for the erection of a new administration building, with adequate laundry and kitchen accommodation. Speaking generally, the New York Hospital is undoubtedly the best administered of those within this city; and its present condition testifies to the ability of the Superintendent (Mr. Geo. B. Ludlam). The grounds are well kept, though not extensive, and the atmosphere within the hospital impresses the visitor with the feeling that everybody is doing the best to make the whole machine run smoothly and efficiently.

